# Fluid intake in term and preterm infants exposed to maternal diabetes

**DOI:** 10.20935/acadnutr8375

**Published:** 2026-06-24

**Authors:** Catherine O. Buck, Veronika Shabanova

**Affiliations:** 1 Department of Pediatrics, Yale School of Medicine, New Haven, CT, USA.

**Keywords:** diabetes in pregnancy, infant growth, infant adiposity, early nutrition, preterm birth

## Abstract

**Introduction:**

Diabetes (DM) in pregnancy is associated with obesity and cardiometabolic abnormalities in childhood. Our prior work has shown that preterm infants exposed to DM in pregnancy have differences in early growth trajectory compared with preterm infants not exposed to DM. The influence of early nutrition on growth patterns in preterm infants exposed to DM in pregnancy is not known. The objective of this study was to examine the influence of DM in pregnancy on early total fluid intake (TFI) and weight change during newborn hospitalization.

**Materials and methods:**

In a prospective cohort of preterm (30–36 weeks) and term infants, nutrition information was obtained from the medical record and adjusted linear mixed effects models used to determine differences in average TFI between DM exposure groups.

**Results:**

Among 149 infants, the DM group (*n* = 50) had less exclusive breastfeeding on day 1. DM group infants had higher TFI (ml/day) in the first postnatal week, which was explained by infant weight. Among late preterm infants, the DM group had accelerated weight between postnatal day 7 and 14 compared with the non-DM group, which correlated with lower TFI for weight (ml/kg/day) on days 3 and 14.

**Conclusions:**

The observed differences in TFI in this cohort may be related to feeding maturity in this at-risk population.

## 1. Introduction

Globally, the proportion of women entering pregnancy with over-weight (body mass index (BMI) > 25 kg/m^2^) or obesity (BMI > 30 kg/m^2^) has more than doubled in recent decades, with current estimates greater than 60% in some countries [1, 2]. This is in parallel to a rise in gestational diabetes mellitus (GDM), which currently affects 14% of pregnancies worldwide, with rates up to 27% in some areas [3–5]. At birth, infants of women with obesity or diabetes in pregnancy have higher birth weight and higher fat mass compared with unexposed infants [6]. In childhood and adolescence, infants of women with these metabolic conditions in pregnancy have twice the risk of cardiometabolic disease, including obesity, insulin resistance, and precursors to cardiovascular disease [7–9]. After birth, ‘catch-down’ growth, or a decrease in weight z-score percentiles in the first months, is inversely related to the risk of later obesity and metabolic abnormalities in term infants of mothers with diabetes and/or obesity [10–12]. In the general population, epidemiologic evidence demonstrates that catch-up growth during infancy relates to an increased risk of obesity in early childhood [13, 14]. Collectively, these findings highlight infancy as a critical window during which alterations in early growth trajectories may shape the long-term cardiometabolic consequences of prenatal exposure to maternal metabolic conditions.

Both diabetes and obesity during pregnancy impart up to a 50% increased risk of preterm birth, most of which is moderate-to-late preterm, or between 32 and 36 weeks’ gestational age [15–17]. Our group’s prior work has demonstrated that preterm infants of women with diabetes in pregnancy have unique growth patterns during the immediate postnatal period [18–20]. While epidemiologic studies have demonstrated the heightened risk of cardiometabolic consequences among children born to women with these metabolic conditions during pregnancy, there is a gap in understanding the role of early nutrition and feeding practices in the development of those chronic disease risks. This study builds upon our group’s prior work to determine if there are differences in prescribed nutrition during newborn hospitalization among infants born to women with and without diabetes in pregnancy. In the newborn period, infants of mothers with obesity or diabetes in pregnancy are poor oral feeders and are more likely to require nasogastric tube feeds [21, 22]. We hypothesized that infants of women with diabetes in pregnancy would have higher total fluid intakes during the first two postnatal weeks, and that these higher fluid intakes would explain variations in early growth in this group.

## 2. Materials and methods

This is a single-center prospective cohort study of moderate-to-late preterm (gestational age (GA) 30 0/7 to 36 6/7 weeks) and term (GA ≥ 37 weeks) infants who were enrolled after birth from the well-baby nursery or neonatal intensive care unit (NICU) between August 2020 and August 2022. This study took place at a single center in New Haven, Connecticut, where all infants < 35 weeks GA are admitted to the level IV NICU. Infants were excluded if the parent did not speak English or Spanish or was <18 years of age. Infants were also ineligible if there was a known congenital or genetic diagnosis which may interfere with growth. The primary aim of this study was to examine infant outcomes related to exposure to obesity and diabetes in pregnancy, and therefore infants born to a birthing parent with diabetes and/or obesity in pregnancy were over-sampled during the enrollment period. Informed consent for participation was obtained from the parents of all subjects involved in the study, and the study was conducted according to the guidelines of the Declaration of Helsinki and approved by the Institutional Review Board of Yale University (protocol code 2000027135; date of approval: 17 March 2020).

During delivery hospitalization, infants were followed for weekly anthropometry measures, including weight, length, and skinfold thicknesses. Weight was obtained by clinical bedside nurses, on a standard scale, with the infant wearing only a diaper. Length was obtained with a length board and a two-person technique by trained research team members [23]. Skinfold thicknesses (SFTs) of the triceps and subscapular regions were obtained in duplicate using Lange skinfold calipers (Seko, Tullytown, PA, USA) by trained research staff members [24]. The duplicate measures were then averaged for the statistical analysis. Mid-upper arm circumference (MUAC) was also measured using a paper measuring tape at the level of the mid-humerus bone [25].

Data regarding pregnancy and infant health characteristics were abstracted from the electronic medical record. The diagnosis of diabetes (DM) in pregnancy including gestational diabetes or pre-pregnancy diabetes mellitus (type 1 or type 2) was abstracted from obstetric team clinical notes and made according to the American College of Obstetricians and Gynecologists [26, 27]. Diabetes treatment was defined as diet-controlled, any insulin, any metformin, or insulin and metformin based on clinical notes and medication administration records. Pre-pregnancy or first-trimester weight and height was obtained from the maternal medical record and classified as pre-pregnancy obesity if the body mass index (BMI) was ≥30 kg/m^2^. Other pregnancy and maternal characteristics included race (Black, White, Asian, or Other/Unknown), ethnicity (Hispanic, non-Hispanic, or other/unknown), age, and delivery mode. Infant characteristics included GA at birth, sex, need for admission or observation in the NICU, hospital length of stay, hypoglycemia during the hospitalization (defined as any value < 40 mg/dl, from the infant problem list), and diagnosis of respiratory distress (i.e., transient tachypnea, respiratory distress syndrome, or retained lung fluid; from the infant problem list).

Infant nutritional information during delivery hospitalization was also obtained from the medical record in the first 2 weeks and on the day prior to discharge. The volume of daily total fluid intake (ml/day) was defined as the recorded fluid intake over a 24 h period, including both intravenous and enteral fluid (both oral and gavage). The 24 h period included volumes received from 07:00 to 06:59 the following calendar day. The calendar day of birth was considered day 0, and day 1 was defined to begin at 07:00 on the calendar day after the day of birth. The infants’ birth weight was used to calculate fluid volumes per kg through day 7, which was done by dividing the total fluid volume (in ml) by the birthweight (in kg). Current weight was used for calculations of fluid volumes in ml/kg after day 7. Additional nutritional information obtained from the medical record included if there were breastfeeding occurrences at the breast in the first two weeks and on the day prior to discharge (yes or no), and the need for parenteral nutrition, intravenous fluids, and/or gavage feedings during the hospital stay (yes or no). Additionally, fortification of the enteral nutrition in the first two weeks and on the day of discharge was also recorded. Unfortified human milk was assumed to be 20 kcal/ounce.

For the statistical analysis, infant health, infant nutrition and growth, and pregnancy characteristics were compared between the two exposure groups of DM versus non-DM in a bivariate analysis. Additionally, these characteristics were compared across GA groups (very preterm (30–31 weeks), moderate preterm (32–33 weeks), late preterm (34–36 weeks), and term (37+ weeks)). Infants were then classified as “exclusively feeding at the breast” for each postnatal day in the first two weeks if all enteral feeds were charted as feeding at the breast. These infants were then excluded from the analysis of fluid outcomes.

For the primary outcome of fluid intake, observed total fluid volume (ml/day) and total fluid volume by weight (ml/kg/day) on postnatal days 1, 2, 3, 7, and 14, and discharge were compared across DM exposure groups using a linear mixed effects model with a random intercept for each infant (random slopes were ruled out using a likelihood ratio test). Based on the prior literature and our initial bivariate analysis, we adjusted the models for infant GA at birth, infant sex, NICU discharge, any need for gavage feeding, any need for total parenteral nutrition, infant length of stay, parental race/ethnicity, and human milk exposure during the hospital stay (yes or no) [18, 22, 28]. We then conducted several secondary analyses to examine differences in the rate of change in fluid volumes and the relationship of fluid intake with change in weight. Using linear contrasts, slopes of change in the fluid volumes were estimated from day 1 to day 3, from day 3 to day 7, and from day 7 to day 14, and compared between groups (DM group—non-DM group). Estimates were summarized with 95% Confidence Intervals (95%CIs).

To examine the relationship of infant fluid intake with early weight trajectory, we evaluated differences in infant growth velocity (weight change in g/day) between birth and postnatal day 3, day 3 to 7, and day 7 to 14 across DM exposure groups. These periods of growth velocity were chosen to mimic the physiologic weight loss pattern which occurs after birth [29]. Using a linear mixed effects model (a random intercept for each infants and no random slopes via an LRT), with knots at postnatal days 3 and 7, we estimated the mean weight (g) with 95%CI over time for each of the DM exposure groups and the between-group difference in the means (DM group—non-DM group). Infants exclusively breastfeeding were excluded, and the models were adjusted for the covariates listed above. The prior literature indicates that early weight change in preterm DM-exposed infants may differ across gestational subgroups [18, 19]. Therefore, we completed an exploratory stratified analysis in which we repeated the models described above among late preterm born infants. In general, all hypotheses’ tests for both the primary and secondary analyses were conducted at the two-sided alpha level of 0.05, and conclusions regarding clinically meaningful effects relied upon effects sizes and 95% CIs [30–32]. All analyses were conducted in SAS Version 9.4 (SAS Institute Inc., Cary, NC, USA).

## 3. Results

Among 595 eligible term and preterm infants born during the study period, 286 (48%) were approached, and 150 (25%) enrolled (Figure S1). One infant was excluded from the analysis due to diagnosis of a major congenital anomaly after initial enrollment. In the final cohort of 149 infants, 99 (66%) were in the non-DM group and 50 (34%) in the DM group. The DM group was approximately half gestational diabetes (54%), and most were born to mothers who required insulin and/or metformin for treatment during pregnancy (66%). Infants in the DM group had higher regional SFT measures at both study enrollment and near the time of discharge (*p* < 0.01 for both; Table 1). Hospital length of stay, infant gestational age, and the diagnoses of respiratory distress and hypoglycemia during the hospital admission were similar between the DM and non-DM groups (Table 1). Overall nutritional intakes were similar between the DM exposure groups, although there were some small differences across time in the use of fortified feeds in the late preterm infants, with non-DM late preterm infants having higher use of fortified feeds compared with the late preterm DM infants (Tables S1 and S2).

Given the range of gestational ages included in the study sample, we examined pregnancy health, infant health, and infant nutritional outcomes across GA subgroups. Overall, 68 (46%) infants were born full term, and 81 (54%) were born preterm (median GA 34.4 weeks). Across GA subgroups (term, late preterm, moderate preterm, and very preterm), the preterm groups were more likely to be multiple-gestation, admitted to and/or discharged from the NICU, and have smaller enrollment and discharge anthropometry measures (Table S3). Compared with the term group, very preterm infants had greater use of parenteral nutrition, IV fluids, and 24 calorie feeds at hospital discharge (Table S4).

**Figure 1 •:**
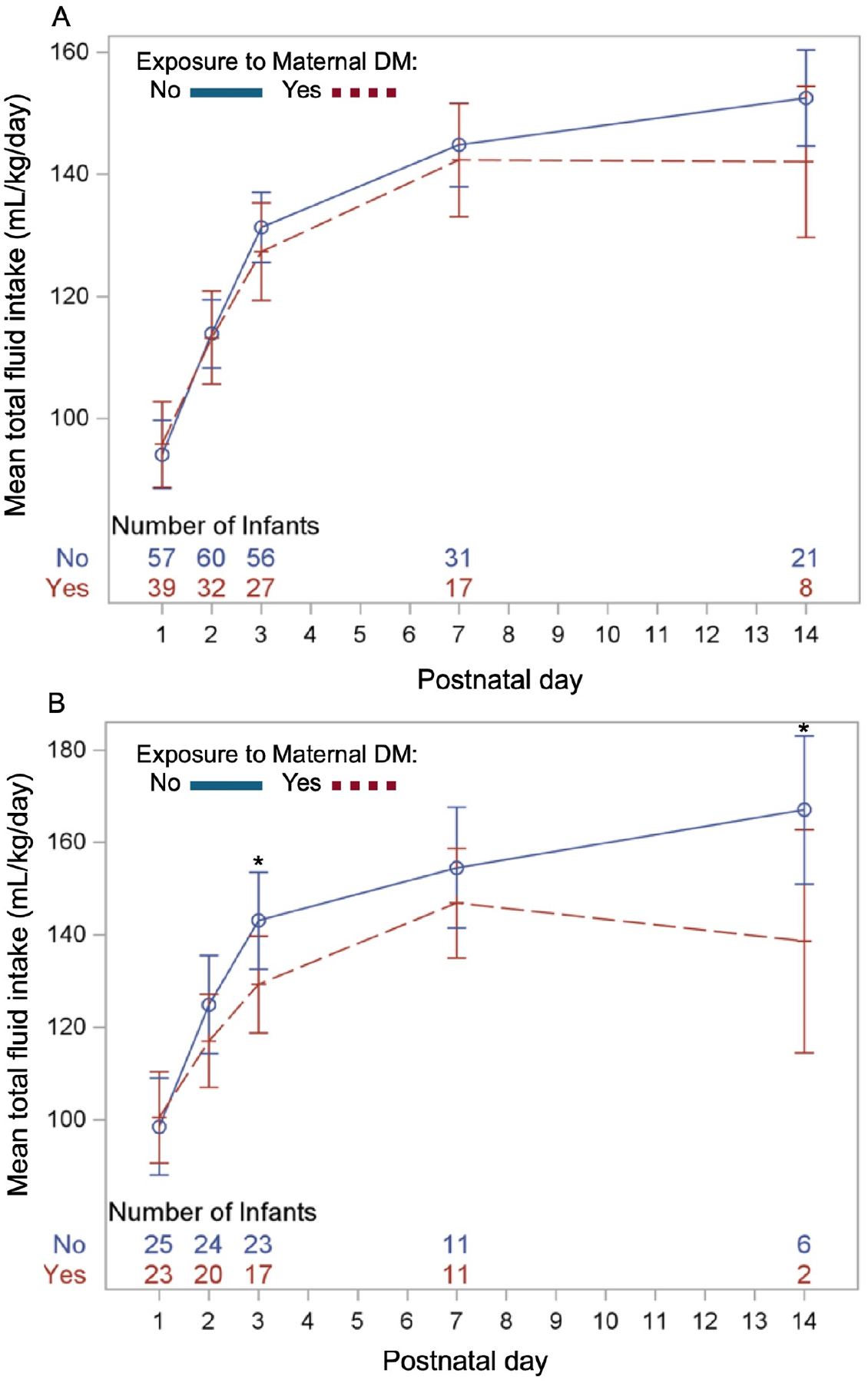
Predicted mean total fluid intake (ml/kg/day) in first 2 weeks by diabetes (DM) in pregnancy exposure in (**A**) the entire cohort and (**B**) in late preterm infants only, after excluding infants who were exclusively breastfeeding. * Denotes difference between groups, *p*-value < 0.05.

Fewer infants in the DM group were exclusively breastfeeding on postnatal day 1 compared with the non-DM group (8% vs. 28%, *p* = 0.01; Figure S2). Among infants not exclusively breastfeeding, the DM group received a higher total fluid volume (ml/day) in the first postnatal week compared with infants in the non-DM group (Table 2). This difference in fluid volume is driven by infant weight, as the volumes were similar across the DM exposure groups when calculated as total volume by weight (ml/kg/day). Overall, infants received approximately 95 ml/kg/day on postnatal day 1, 130 ml/kg/day on postnatal day 3, and 140 ml/kg/day on postnatal day 7 (Table 2). The rate of the increase in fluid intake over time was highest from birth to day 3, but was overall not different between the DM exposure groups (Table 2; Figure 1A).

In the stratified analysis of late preterm infants, DM group late preterm infants had higher total prescribed fluid on day 1, but lower total fluid per weight on days 3 and 14 compared with late preterm infants in the non-DM group (Table 3; Figure 1B). In DM group late preterm infants, there were also differences in the rate of change in fluids from birth to day 3 compared with late preterm non-DM infants (Table 3). These differences did not persist after day 3.

**Figure 2 •:**
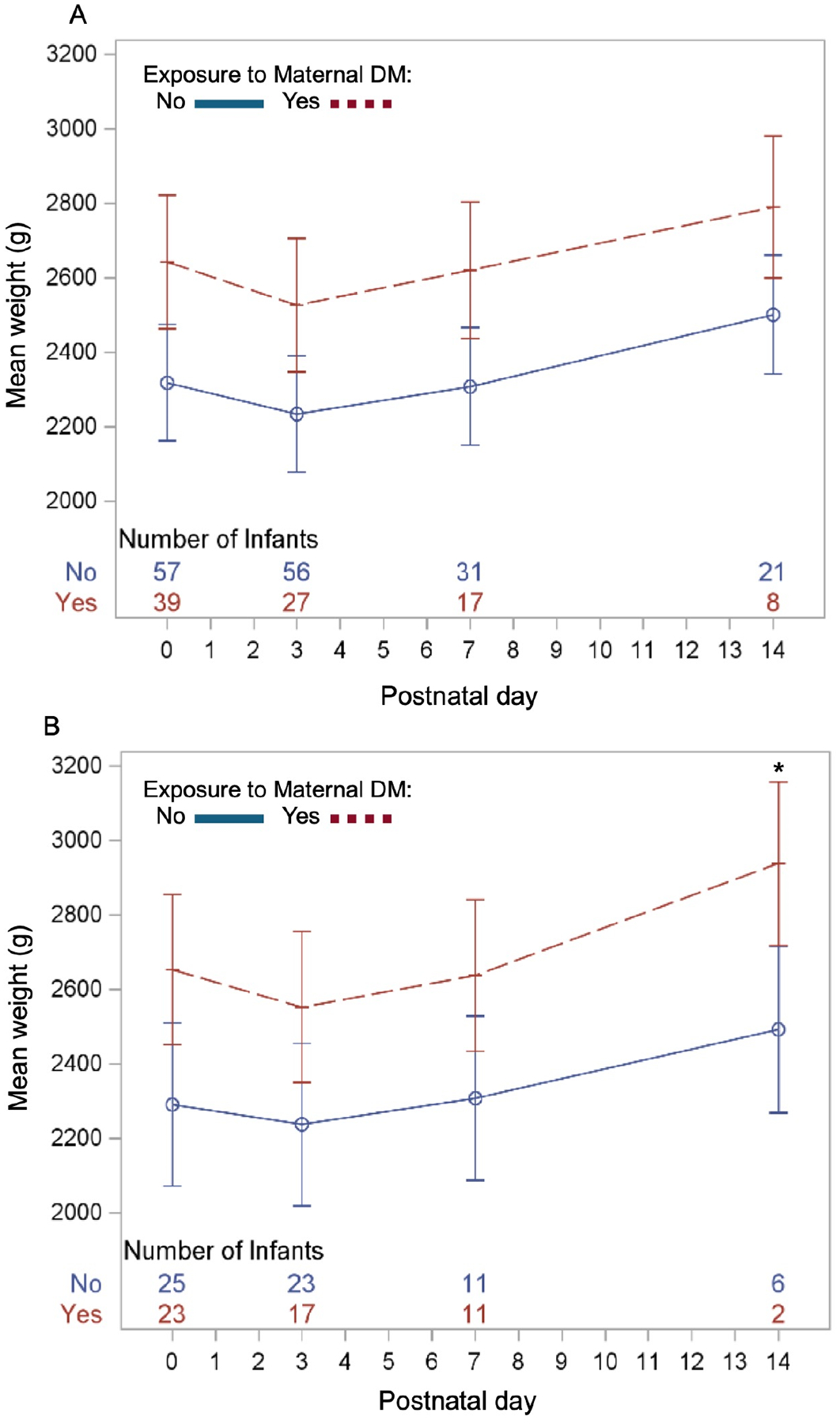
Predicted mean weight over the first 2 weeks by diabetes (DM) in pregnancy exposure in (**A**) the entire cohort and (**B**) in late preterm infants only, after excluding infants who were exclusively breastfeeding. * Denotes difference between groups, *p*-value < 0.05.

In this cohort, change in weight in the first postnatal week was not meaningfully different across DM exposure groups (Table S5; Figure 2A). In the sensitivity analysis of late preterm infants only (born 34 to 36 weeks GA), infants in the DM group had more 16.5 g/day more rapid weight gain (95% CI: 0.5, 32.4) from postnatal day 7 to 14 compared with the late preterm non-DM group (Table S5; Figure 2B). This period of greater weight gain corresponded to the period of lower fluid intake among late preterm DM group infants.

## 4. Discussion

In this single-center prospective cohort study, we found that term and preterm infants born to women with diabetes in pregnancy had overall similar nutritional exposures compared with infants in the unexposed group, including total human milk exposure, need for any supplemental calories during the hospital stay, gavage feedings, and prescribed total daily fluid intakes over the first two postnatal weeks. While total fluid volumes (ml per day) were overall higher in the DM-exposed group, this was due to higher infant birthweight in the DM group, indicated by a lack of meaningful difference after calculation of fluids per kilogram weight. In our exploratory subgroup analysis, late preterm DM-exposed infants had overall lower total fluid volume intakes in the second week, during which time growth velocity was higher in that group. While there were not meaningful differences in the use of supplemental calories, it is not known if there were differences in total energy intake across groups. In general, this is a subgroup of preterm infants for which there are limited data to support specific nutritional approaches. In the context of perinatal exposures, including metabolic health in pregnancy, our results highlight the need for additional studies to determine the optimal nutritional strategy, including approaches to precision nutrition, to support growth in this group.

Although our study was not able to differentiate enteral fluids from intravenous fluids when calculating total fluid intake, intravenous fluid requirements (yes or no) were not meaningfully different across this cohort. Neither was the diagnosis of hypoglycemia, which is expected to be the most common clinical reason for intravenous fluid requirements in these infants. While our results did not reveal confounding by these variables, future studies are required to further examine the role of enteral versus intravenous fluid intakes in weight trajectories in this subgroup of preterm infants. In our unit, it is standard that 60 to 80 ml/kg/day of intravenous fluid is started when an infant presents with critical hypoglycemia to maintain an adequate glucose infusion rate.

There is likely practice variability across neonatal intensive care units regarding the approach to hypoglycemia treatment. One prior study in infants born ≥ 35 weeks’ gestation found that length of stay and glucose variability were decreased when treatment of hypoglycemia with intravenous fluids started at 30 ml/kg compared with 60 ml/kg [33]. Additionally, a recent larger clinical trial of early full enteral feeds compared with progressive enteral feeding in infants born 30-to-32 weeks’ gestational age demonstrated similar rates of hypoglycemia across groups [34]. However, the role of these different clinical approaches to early fluid management on early growth in this subgroup of infants remains unclear. This is a critical area of study given the data to suggest that early growth, particularly early accelerated growth, is directly linked to subsequent chronic disease [13, 35]. Clinical trials are required to further examine the direct effect of alterations in early fluid with weight loss patterns in this group of infants.

In our study, early exclusive breastfeeding was lower in infants born to women with diabetes in pregnancy compared with those born to women without diabetes. While we did not assess intention to breastfeed, this finding is in line with other studies showing that women with gestational diabetes have lower rates of exclusive breastfeeding and shorter duration of breastfeeding compared with women who did not have diabetes in pregnancy [28, 36]. Neonatal intensive care unit admission and mother–infant dyad separation may have a role in this finding. It is also possible that nutritional management, in which there is a ‘fear’ of hypoglycemia, plays a role in the amount and type of enteral nutrition that is prescribed [37]. Additionally, infants born to women with diabetes in pregnancy are often labeled as poor oral feeders. Some studies have demonstrated greater need for nasogastric tube feeds and slower attainment of full oral feeds in infants born to women with diabetes in pregnancy [21, 22]. In the present study, we did not observe meaningful differences in the need for nasogastric feeds by diabetes exposure group. This may be due to sample size and the range of gestational ages included in our cohort. Emerging evidence suggests that there are alterations in early sucking patterns in term infants of diabetic mothers [38]. However, there is also evidence that some of these early feeding patterns may be related to infant body composition, such that higher fat mass relates to lower hunger signaling [22, 39]. This leads to the question, what is the right feeding target in this group of infants?

Epidemiologic studies have shown that infants born large for gestational age often exhibit a pattern of ‘catch-down’ growth, or a decrease in weight z-score percentiles in the first months, and that this early weight loss is in turn inversely related to the risk of later obesity and metabolic abnormalities in this group [10–12]. On the contrary, accelerated growth in large-for-gestational-age infants and in infants born to women with metabolic conditions in pregnancy imparts even higher risk for long-term metabolic sequalae [40, 41]. In the present study, overall weight trends followed the expected pattern of early loss through postnatal day 3 followed by gain through discharge, without meaningful differences between the diabetes-exposed and -un-exposed groups. While this cohort study did not examine growth past hospital discharge, we did find that late preterm infants born to women with diabetes had accelerated weight gain in the second week, a pattern which is associated with risk of obesity in other populations [11, 13]. These findings are also in line with our prior work, in which late preterm DM-exposed infants had faster weight gain through age 2 years compared with un-exposed preterm infants [19]. Additional studies which focus on longitudinal outcomes related to these early differences in both nutritional intake and growth patterns will elucidate what the optimal fluid target is. For example, should lower total fluid intake be prescribed so that some degree of early weight loss is achieved? Targeting nutritional intakes based on expected growth potential in small-for-gestational-age infants has been demonstrated to be a feasible precision nutrition strategy in one recent pilot clinical trial [42].

Strengths of this study include its prospective design, modest sample size, and inclusion of infants from an overall understudied group regarding growth and nutrition research. The cohort includes an understudied population of preterm infants, and over-sampling of infants born to women with diabetes in pregnancy has enhanced our ability to detect exposure-related differences in early nutritional practices. Additionally, the dataset includes rich, high-resolution nutritional data including prescribed total fluid intakes during NICU hospitalization. The main weaknesses of this study include its single-center design and the lack of follow-up data beyond the initial hospitalization. The dataset used for this analysis could not distinguish between volume of enteral feeds and volume of intravenous feeds, and the exclusion of exclusively breastfed infants may have introduced selection bias. There is also the potential that unit-protocols specific to our center drive the variability in fluid prescriptions observed in this study [40, 41]. Protocols for treatment of hypoglycemia vary across institutions, and this may limit the generalizability of our results [43]. Additionally, there is the potential for missing data and/or some factors which were not able to be assessed in this cohort, including caloric intake/density of individual feedings, data regarding control or treatment of diabetes prior to and during pregnancy, and lactation support variables/assessments of pre- and post-breastfeeding weights. We also acknowledge the heterogeneity of phenotypes represented in the diabetes-exposed group in this study. This study’s design, with enrollment after birth, did not allow for robust phenotyping of maternal metabolic health prior to and in early pregnancy, and given the small sample size, it did not allow for further subgrouping of diabetes phenotype. Additional studies are required to understand the nuances of diabetes type and control with these neonatal feeding and growth outcomes.

## 5. Conclusions

In summary, in this prospective cohort of term and preterm infants, those born to women with diabetes in pregnancy had overall similar nutritional exposures during newborn hospitalization compared with infants born to women without diabetes in pregnancy. In subgroup analysis, late preterm infants born to women with diabetes in pregnancy had subtle differences in fluid intake patterns which corresponded to more rapid weight gain in the second postnatal week when compared with the late preterm un-exposed group. It is possible that these results reflect feeding dysmaturity in this group, and additional studies are required to examine the long-term outcomes related to differences in early nutritional intakes in this population of infants. A clearer understanding of how early feeding practices shape early growth trajectories in this population is essential for informing precision nutrition strategies aimed at improving long-term metabolic health in high-risk populations.

## Supplementary Material

Supplementary materials

The supplementary materials are available at https://doi.org/10.20935/AcadNutr8375.

## Figures and Tables

**Table 1 •:** Pregnancy and infant health characteristics by diabetes mellitus (DM) in pregnancy exposure, *N* = 149.

Characteristic	Non-DM group *n* = 99	DM group *n* = 50	*p*-value *
Maternal age (years)	31.9 (5.3)	33.7 (5.5)	0.04
**Infant race:**			0.37
Black	15 (15%)	9 (18%)	
Other	11 (11%)	9 (18%)	
White	73 (74%)	32 (64%)	
**Infant ethnicity:**			0.03
Hispanic	28 (28%)	17 (34%)	
Non-Hispanic	71 (72%)	30 (60%)	
Unknown	0 (0%)	3 (6%)	
Maternal BMI (kg/m^2^) ^a^	30.0 (8.7)	32.9 (7.9)	0.05
Pre-eclampsia	16 (16%)	10 (20%)	0.55
**Diabetes type:**			N/A
Gestational	N/A	27 (54%)	
Type 1		9 (18%)	
Type 2		14 (28%)	
**Diabetes medications:**			N/A
None	N/A	17 (34%)	
Metformin only		1 (2%)	
Insulin only		27 (54%)	
Insulin and metformin		5 (10%)	
C-section delivery	56 (57%)	32 (64%)	0.38
Gestational age (weeks)	36.6 (3.1)	36.2 (2.3)	0.43
**Birthweight category:** ^b^			0.08
SGA	11 (11%)	1 (2%)	
AGA	78 (79%)	38 (76%)	
LGA	10 (10%)	11 (22%)	
Multiple gestation	25 (26%)	3 (6%)	0.004
Female infant sex	40 (40%)	22 (44%)	0.67
NICU observation and/or admission	87 (88%)	49 (98%)	0.06
NICU discharge	68 (69%)	33 (66%)	0.87
Length of stay (days)	13.2 (18.0)	10.1 (10.6)	0.19
Hypoglycemia	43 (43%)	29 (58%)	0.09
Respiratory distress diagnosis	52 (53%)	29 (58%)	0.53
**Birth anthropometry:** ^c^			
Weight (kg)	2.63 (2.06, 3.30)	2.93 (2.49, 3.41)	0.06
Length (cm)	47.5 (44.5, 50.8)	48.3 (46.0, 51.4)	0.26
Head circumference (cm)	32.5 (30.5, 34.5)	32.8 (30.0, 34.0)	0.66
Mid arm circumference (cm)	9.0 (8.0, 10.0)	9.8 (8.5, 11.0)	0.43
Triceps SFT (mm)	4.0 (3.0, 5.0)	5.0 (4.0, 6.5)	<0.001
Subscapular SFT (mm)	4.0 (3.0, 4.5)	4.5 (3.5, 5.5)	<0.001
**Near-discharge anthropometry:** ^c^			
Weight (kg)	2.80 (2.31, 3.19)	2.97 (2.47, 3.40)	0.19
Length (cm)	48.9 (46.0, 50.8)	49.0 (47.5, 52.0)	0.37
Head circumference (cm)	33.3 (32.0, 35.0)	33.0 (31.5, 34.0)	0.15
Mid arm circumference (cm)	9.5 (9.0, 11.0)	10 (9.5, 10.0)	0.85
Triceps SFT (mm)	4.0 (3.0, 5.0)	4.5 (4.0, 5.8)	0.005
Subscapular SFT (mm)	4.0 (3.0, 5.0)	5 (4.0, 6.5)	0.007
PMA at near-discharge measures (weeks)	37.1 (35.7, 39.3)	36.8 (35.4, 37.8)	0.17

DM (diabetes); NICU (newborn intensive care); BMI (body mass index); PMA (post menstrual age); SFT (subscapular skinfold thickness); SGA (small-for-gestational age); AGA (appropriate-for-gestational age); LGA (large-for-gestational age). Expressed as *n* (%) or mean (standard deviation, sd), unless otherwise noted.

* chi-square test for categorical variables and the Wilcoxon Rank Sum test for continuous variables.

a Calculated from pre-pregnancy or first-trimester weight (earliest available) and height.

b Calculated from Fenton reference curves based on sex and gestational age.

c Expressed as median (interquartile range, IQR).

**Table 2 •:** Adjusted ^1^ predicted mean total fluid volume and change in fluid volumes over time during the first two postnatal weeks by diabetes (DM) in pregnancy exposure.

	Non-DM group β (95% CI)	DM group β (95% CI)	*p*-value ^2^
**Total prescribed fluid volume (ml)**			
Day 1 (*n* = 96)	207.4 (185.64, 229.13)	242.4 (217.9, 266.9)	0.02
Day 2 (*n* = 92)	241.8 (220.2, 263.4)	273.5 (247.8, 299.1)	0.04
Day 3 (*n* = 83)	275.5 (253.8, 297.1)	305.4 (278.9, 331.9)	0.05
Day 7 (*n* = 48)	312.5 (288.4, 336.5)	349.7 (320.4, 379.0)	0.04
Day 14 (*n* = 29)	358.2 (332.1, 384.3)	380.7 (344.3, 417.1)	0.31
Discharge (*N* = 149)	338.2 (269.7, 406.7)	414.4 (349.8, 470.0)	0.12
**Change per day in total prescribed fluid volume (ml)**			
Birth to day 3	33.9 (26.6, 41.3)	31.6 (21.7, 41.4)	0.70
Day 3 to 7	9.3 (5.0, 13.6)	11.0 (5.2, 16.8)	0.63
Day 7 to 14	6.5 (3.4, 9.7)	4.4 (−0.5, 9.2)	0.46
**Total fluid per weight (ml/kg)**			
Day 1 (*n* = 96)	94.1 (88.5, 99.7)	95.8 (88.8, 102.8)	0.71
Day 2 (*n* = 92)	113.9 (105.6, 120.9)	113.3 (105.6, 120.9)	0.89
Day 3 (*n* = 83)	131.3 (125.6, 137.1)	127.4 (119.4, 135.4)	0.43
Day 7 (*n* = 48)	144.9 (138.0, 151.7)	142.3 (133.1, 151.6)	0.66
Day 14 (*n* = 29)	152.5 (144.6, 160.4)	142.1 (129.7, 154.4)	0.16
Discharge (*N* = 149)	136.0 (112.8, 159.2)	132.1 (110.2, 154.0)	0.81
**Change per day in total fluid per weight (ml/kg)**			
Birth to day 3	18.6 (15.7, 21.5)	15.9 (12.1, 19.7)	0.27
Day 3 to 7	3.3 (1.6, 4.9)	3.6 (1.3, 5.8)	0.84
Day 7 to 14	1.1 (−0.1, 2.3)	-0.03 (−1.9, 1.8)	0.32

DM (diabetes).

1 Results are from generalized linear mixed effects models with random intercept for each infant, adjusted for gestational age at birth, infant sex, parental race, neonatal intensive care discharge, need for any gavage feeding, hospital length of stay, need for total parenteral nutrition and any breastmilk during hospital stay.

2 *p*-value of the between-group difference, DM group—non-DM group.

**Table 3 •:** Stratified analysis of adjusted ^1^ predicted mean total fluid volume in the first two postnatal weeks by diabetes in pregnancy exposure status among only late preterm infants (*n* = 59).

	Non-DM group β (95% CI)	DM group β (95% CI)	*p*-value ^2^
**Total prescribed fluid volume (ml)**			
Day 1 (*n* = 48)	225.1 (196.4, 253.8)	262.1 (235.3, 288.9)	0.026
Day 2 (*n* = 45)	276.1 (247.4, 305.0)	299.9 (272.7, 327.1)	0.16
Day 3 (*n* = 43)	316.1 (287.6, 344.6)	317.2 (289.0, 345.4)	0.95
Day 7 (*n* = 45)	344.9 (310.1, 379.6)	373.2 (341.7, 404.7)	0.19
Day 14 (*n* = 53)	408.2 (366.1, 450.3)	418.2 (356.3, 480.2)	0.78
**Change per day in total prescribed fluid volume (ml)**			
Birth to day 3	45.6 (34.5, 56.8)	28.0 (15.7, 40.3)	0.04
Day 3 to 7	6.6 (−0.5, 13.8)	13.0 (5.9, 20.2)	0.21
Day 7 to 14	9.1 (3.2, 15.1)	6.5 (−2.3, 15.3)	0.62
**Total fluid per weight (ml/kg)**			
Day 1 (*n* = 48)	98.5 (88.0, 109.1)	100.5 (90.7, 110.4)	0.74
Day 2 (*n* = 45)	124.5 (114.3, 135.5)	117.1 (107.0, 127.1)	0.21
Day 3 (*n* = 43)	143.1 (132.6, 153.6)	129.3 (118.8, 139.7)	0.036
Day 7 (*n* = 45)	154.6 (141.5, 167.6)	146.9 (135.1, 158.7)	0.35
Day 14 (*n* = 53)	167.0 (151.0, 183.1)	138.7 (114.5, 162.8)	0.046
**Change per day in total fluid per weight (ml/kg)**			
Birth to day 3	22.4 (18.0, 26.8)	14.5 (9.6, 19.3)	0.02
Day 3 to 7	2.5 (−0.3, 5.3)	4.2 (1.4, 7.0)	0.39
Day 7 to 14	1.8 (−0.6, 4.1)	-1.2 (−4.7, 2.3)	0.16

DM (diabetes).

1 Results are from generalized linear mixed effects models with random intercept for each infant, adjusted for gestational age at birth, infant sex, parental race, neonatal intensive care discharge, and any breastmilk during hospital stay.

2 *p*-value of the between-group difference, DM group—non-DM group.

## Data Availability

The data supporting the findings of this publication can be made available upon request.
