# Supplementary material for: Fluid intake in term and preterm infants exposed to maternal diabetes
Source: Acad Nutr Diet. Author manuscript; Available in PMC 2026 Jul 5. (PMC13332685; doi:10.20935/acadnutr8375)
Supplement: Supplementary materials [file NIHMS2190905-supplement-Supplementary_materials.pdf]

Supplementary materials

Table S1. Hospital nutritional information by diabetes (DM) in pregnancy exposure, *N* = 149.

	Non-DM group *n* = 99	DM-group *n* = 50	*p*-value *
**Overall**			
Any breastmilk during hospital stay	87 (88%)	41 (82%)	0.33
Any parenteral nutrition	11 (11%)	04 (8%)	0.55
Any intravenous fluids	48 (49%)	24 (48%)	0.96
Any gavage feeds in first 14 days	48 (48%)	22 (44%)	0.84
Receiving breastmilk at discharge	79 (80%)	36 (72%)	0.28
**Prescribed feeds at discharge:**			
20 kcal/ounce	68 (59%)	33 (66%)	0.42
22 kcal/ounce	16 (26%)	13 (26%)	
24 kcal/ounce	15 (15%)	4 (8%)	
**Across postnatal day**			
**Intravenous fluids**			
Day 1 (*N* = 149)	44/99 (44%)	23/50 (46%)	0.85
Day 2 (*n* = 133)	28/86 (33%)	17/47 (36%)	0.67
Day 3 (*n* = 108)	12/70 (17%)	7/38 (18%)	0.87
Day 7 (*n* = 67)	2/44 (5%)	2/23 (9%)	0.49
Day 14 (*n* = 39)	1/29 (3%)	1/10 (10%)	0.12
**Gavage feeds**			
Day 1 (*N* = 149)	42/99 (42%)	18/50 (36%)	0.45
Day 2 (*n* = 133)	42/86 (49%)	19/47 (40%)	0.35
Day 3 (*n* = 108)	40/70 (57%)	17/38 (45%)	0.22
Day 7 (*n* = 67)	35/44 (80%)	16/23 (70%)	0.36
Day 14 (*n* = 38)	20/29 (69%)	6/9 (67%)	0.89
**Fortified feeds (≥22 kcal/ounce)**			
Day 1 (*N* = 149)	27/99 (27%)	18/50 (36%)	0.27
Day 2 (*n* = 133)	29/86 (34%)	15/47 (32%)	0.45
Day 3 (*n* = 108)	28/70 (40%)	15/38 (39%)	0.96
Day 7 (*n* = 67)	36/44 (82%)	15/23 (65%)	0.10
Day 14 (*n* = 39)	25/29 (86%)	8/10 (80%)	0.64

DM (diabetes mellitus in pregnancy). Expressed as *n* (%) or mean (standard deviation, sd), unless otherwise noted. * chi-square test for categorical variables and the Wilcoxon Rank Sum test.

Table S2. Detailed nutritional information by postnatal day and diabetes (DM) in pregnancy exposure among late preterm infants (34 to 36 weeks' gestational age, *n* = 59).

	**Day 1 (*n* = 59)**	**Day 2 (*n* = 57)**	**Day 3 (*n* = 55)**	**Day 7 (*n* = 36)**	**Day 14 (*n* = 14)**
	**IV fluid exposure**				
Non-DM group	15/34 (44%)	13/32 (41%)	3/32 (9%)	0/21 (0%)	0/11 (0%)
DM group	13/25 (52%)	9/25 (36%)	5/23 (22%)	1/15 (7%)	0/3 (0%)
p-value*	0.549	0.722	0.20	0.23	N/A
	**Fortified feeds (≥22 kcal/ounce)**				
Non-DM group	18/34 (53%)	20/32 (63%)	19/32 (59%)	20/21 (95%)	9/11 (82%)
DM group	12/25 (48%)	10/25 (40%)	9/23 (39%)	8/15 (53%)	2/3 (67%)
p-value*	0.71	0.06	0.17	0.005	0.74

DM (diabetes mellitus in pregnancy); * chi-square test for categorical variables. Data present as *n* (%).

Table S3. Pregnancy and infant health characteristics by gestational age subgroup, *N* = 149.

Characteristic	Very preterm	Moderate preterm	Late preterm	Full term	*p*-value*
	***n* = 9**	***n* = 13**	***n* = 59**	***n* = 68**	
Maternal age (years)	31.4 (3.3)	32.1 (4.4)	32.6 (5.9)	32.4 (5.4)	0.94
**Infant race**					
Black	1 (11)	4 (31)	6 (10)	13 (19)	0.21
Other	1 (11)	2 (15)	5 (8)	12 (18)	
White	7 (78)	7 (54)	48 (81)	43 (63)	
**Infant ethnicity**					
Hispanic	3 (33)	1 (8)	18 (31)	23 (34)	0.55
Non-Hispanic	6 (67)	12 (92)	40 (68)	43 (63)	
Unknown	0 (0)	0 (0)	1 (2)	2 (3)	
Maternal BMI (kg/m^2) [a]	28.6 (7.5)	28.9 (6.4)	31.1 (8.7)	31.6 (8.9)	0.60
Diabetes in pregnancy	02 (22)	04 (31)	25 (42)	19 (28)	0.32
C-section delivery	05 (56)	09 (69)	38 (64)	36 (53)	0.50
Multiple gestation	05 (56)	02 (15)	18 (31)	03 (5)	<0.001
Female infant sex	05 (56)	04 (31)	27 (46)	26 (38)	0.55
NICU observation or admission	09 (100)	13 (100)	57 (97)	57 (84)	0.05
NICU discharge	09 (100)	13 (100)	44 (75)	35 (52)	<0.001
Length of stay (days)					<0.001
mean (sd)	48.4 (25.8)	25.2 (8.0)	12.1 (13.8)	4.9 (6.5)	<0.001
median (25th–75th)	39.0 (30.0, 71.0)	26.0 (18.0, 28.0)	10.0 (5.0, 13.0)	3.0 (2.0, 5.0)	
**Birth anthropometry:** [b]					
Weight (kg)	1.47 (1.33, 1.78)	1.91 (1.68, 2.31)	2.43 (2.06, 2.79)	3.37 (3.08, 3.60)	<0.001
Length (cm)	40.0 (39.0, 45.0)	44.0 (41.5, 45.0)	46.0 (44.5, 48.5)	51.0 (49.5, 52.8)	
Head circumference (cm)	29.0 (27.0, 32.0)	30.0 (27.5, 31.0)	31.5 (30.0, 32.5)	34.2 (33.0, 35.3)	
Mid arm circumference (cm)	7.1 (6.4, 7.8)	7.5 (6.0, 8.5)	9.0 (8.5, 10.0)	10.0 (9.5, 11.5)	
Triceps SFT (mm)	2.4 (2.0, 3.1)	3.0 (2.3, 4.0)	4.0 (3.0, 5.0)	5.0 (4.0, 5.5)	
Subscapular SFT (mm)	2.3 (2.0, 2.9)	3.0 (2.0, 3.5)	4.0 (3.0, 5.0)	4.3 (3.8, 5.0)	
**Near-discharge anthropometry:** [b]					
Weight (kg)	2.62 (2.32, 2.82)	2.53 (2.22, 2.98)	2.47 (2.20, 2.78)	3.18 (2.95, 3.63)	<0.001
Length (cm)	48.0 (46.5, 50.0)	46.5 (45.0, 48.0)	47.3 (45.0, 49.0)	51.0 (49.5, 53.0)	<0.001
Head circumference (cm)	34.0 (33.0, 34.5)	32.0 (31.3, 33.8)	32.0 (31.0, 33.0)	34.3 (33.0, 35.5)	<0.001
Mid arm circumference (cm)	8.0 (6.5, 9.0)	9.0 (8.0, 10.0)	9.0 (8.5, 10.0)	10.3 (9.5, 11.5)	<0.001
Triceps SFT (mm)	3.0 (2.0, 3.0)	4.8 (3.0, 5.5)	4.0 (3.0, 5.5)	5.0 (4.0, 5.5)	0.006
Subscapular SFT (mm)	3.0 (2.0, 3.3)	4.0 (3.0, 5.0)	4.0 (3.0, 5.0)	4.4 (4.0, 5.5)	0.008
PMA at near-discharge measures	33.9 (32.7, 35.1)	35.1 (34.6, 35.9)	35.9 (35.1, 36.7)	39.1 (38.4, 40.0)	<0.001

Very preterm (<32 weeks), Moderate preterm (32 to 33 weeks), Late preterm (34–36 weeks), Full term (≥ 37 weeks); NICU (newborn intensive care); BMI (body mass index), PMA (post menstrual age), SFT (subscapular skin fold thickness). Expressed as *n* (%) or mean (standard deviation, sd), unless otherwise noted; * chi-square test for categorical variables and the Wilcoxon Rank Sum test. [a] Defined as pre-pregnancy or first trimester weight; [b] Expressed as median (25th–75th).

Table S4. Hospital nutritional information by gestational age subgroup, $N = 149$.

Characteristic	Very preterm $n = 9$	Moderate preterm $n = 13$	Late preterm $n = 59$	Full term $n = 68$	p-value *
Any breastmilk during hospital stay	09 (100)	13 (100)	47 (80)	59 (87)	0.18
Any parenteral nutrition	08 (89)	05 (38)	01 (2)	01 (2)	<0.001
Any intravenous fluids	09 (100)	12 (92)	30 (51)	21 (31)	<0.001
Gavage feeds in first 14 days	09 (100)	13 (100)	35 (59)	13 (19)	<0.001
Receiving breastmilk at discharge	07 (78)	11 (85)	40 (68)	57 (84)	0.17
**Prescribed calories at discharge:**					
20 kcal/ounce	(0)	0 (0)	25 (42)	66 (97)	<0.001
22 kcal/ounce	1 (11)	10 (77)	27 (46)	1 (1.5)	
24 kcal/ounce	8 (89)	3 (23)	7 (12)	1 (1.5)	

* Chi-squared test.

Table S5. Estimated difference (DM group—non-DM group) in weight gain velocity (g/day) over the first 2 postnatal weeks.

Weight gain velocity (g/day)	Total cohort $n = 105$	Late preterm infants only $n = 52$
	DM group—non-DM group β (95% CI)	DM group—non-DM group β (95% CI)
Birth to day 3	−10.7 (−28.2, 6.9)	−15.2 (−31.2, 0.78)
Day 3 to 7	5.1 (−10.5, 20.6)	3.5 (−11.4, 18.5)
Day 7 to 14	−3.3 (−16.6, 9.9)	16.5 (0.5, 32.4) *

* p-value < 0.05. Infants excluded if exclusively breastfeeding. DM (diabetes mellitus in pregnancy)

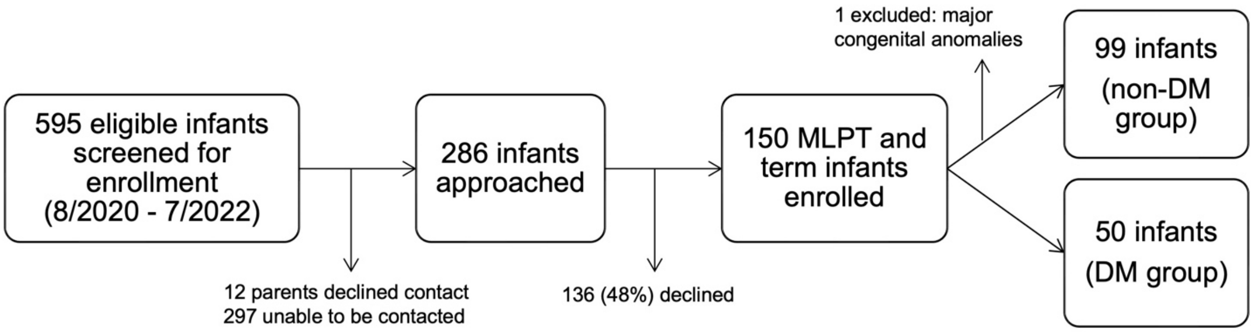

Figure S1. Study flow diagram. MLPT (moderate to late preterm); DM (diabetes mellitus in pregnancy).

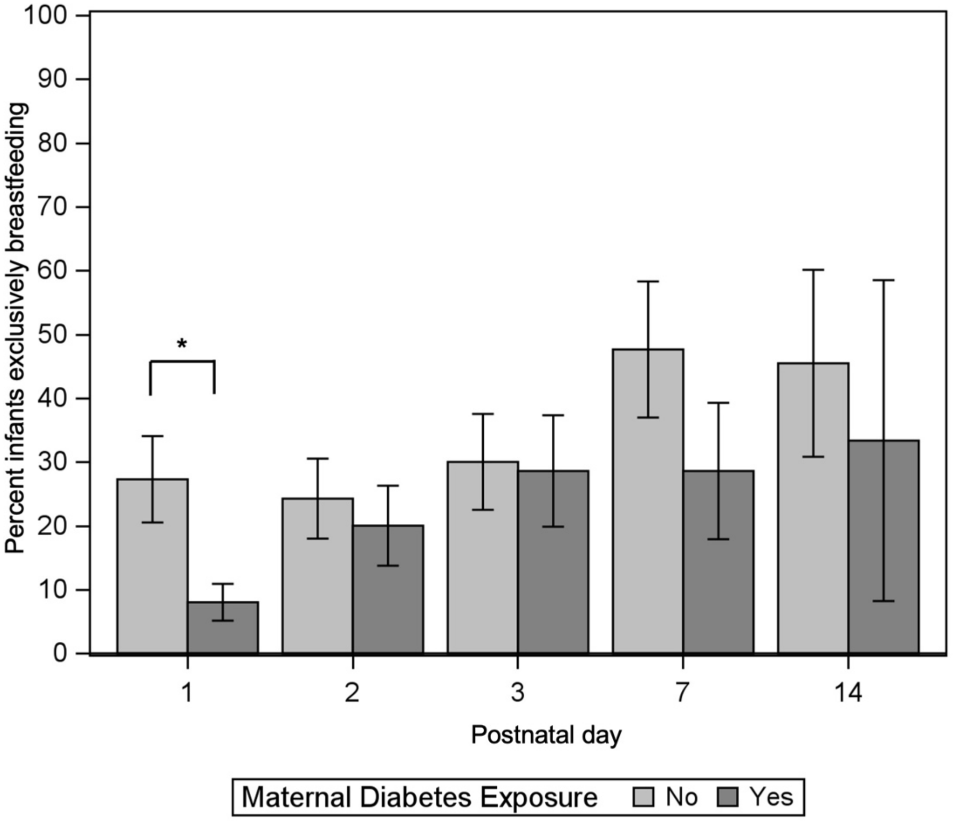

Figure S2. Proportion of infants exclusively breastfeeding in the first 2 postnatal weeks according to diabetes (DM) in pregnancy exposure. Error bars represent standard deviation, *represents chi^2 test *p*-value <0.05.
